# Solitary glandular papilloma of the peripheral lung: a report of two cases

**DOI:** 10.1186/1477-7819-12-149

**Published:** 2014-05-19

**Authors:** Kaoru Kaseda, Hirotoshi Horio, Masahiko Harada, Tsunekazu Hishima

**Affiliations:** 1 Department of Thoracic Surgery, Tokyo Metropolitan Cancer and Infectious Diseases Center Komagome Hospital, 3-18-22, Honkomagome Bunkyo-ku, Tokyo 113-8677, Japan; 2 Department of Pathology, Tokyo Metropolitan Cancer and Infectious Diseases Center Komagome Hospital, Tokyo, Japan; 3 Department of Thoracic Surgery, Sagamihara Kyodo Hospital, Kanagawa, Japan

**Keywords:** Solitary papilloma, Glandular papilloma, Surgical resection

## Abstract

Solitary papilloma of the lung is thought to be a rare benign epithelial tumor, and complete surgical resection is currently the standard treatment for this pathology. However, some cases of papilloma have reportedly shown malignant potential. We report two cases of solitary glandular papilloma of the peripheral lung that were treated by thoracoscopic partial resection. The first patient presented with a nodular lesion in the lower lobe of the left lung that was detected on *a* follow-up chest computed tomography *(CT) scan* after treatment for laryngeal cancer. Partial lung resection was performed by video-assisted thoracoscopic surgery. In the second patient, a nodular lesion was incidentally identified in the lower lobe of the left lung during a health check-up. Partial lung resection was again performed by video-assisted thoracoscopic surgery. *The p*ostoperative course in both cases was uneventful, and no recurrences have been observed as of 44 months and 41 months postoperatively, respectively. To the best of our knowledge, malignant transformation has been reported both with the squamous type and the mixed type of solitary papilloma of the lung. The glandular variant has shown no tendency toward local recurrence after local excision and has no apparent malignant potential. Local excision is thus recommended for solitary glandular papilloma in order to preserve pulmonary function.

## Background

Solitary pulmonary papilloma is a rare neoplasm that is usually derived from bronchial surface epithelium and forms an endobronchial tumor [1-3]. Solitary pulmonary papillomas are subclassified into three categories according to histological type: squamous cell papilloma*,* glandular papilloma*,* and mixed squamous cell and glandular papilloma (mixed papilloma) [2]. Of these, glandular papilloma of the peripheral lung is uncommon, with only 20 cases reported in the English literature [2,4-7]. The clinicopathologic features thus remain unclear. *Here w*e report two cases of solitary glandular papilloma of the peripheral lung, and discuss the clinical implications of surgery for *this*.

## Case presentation

### Case 1

**Figure 1:**
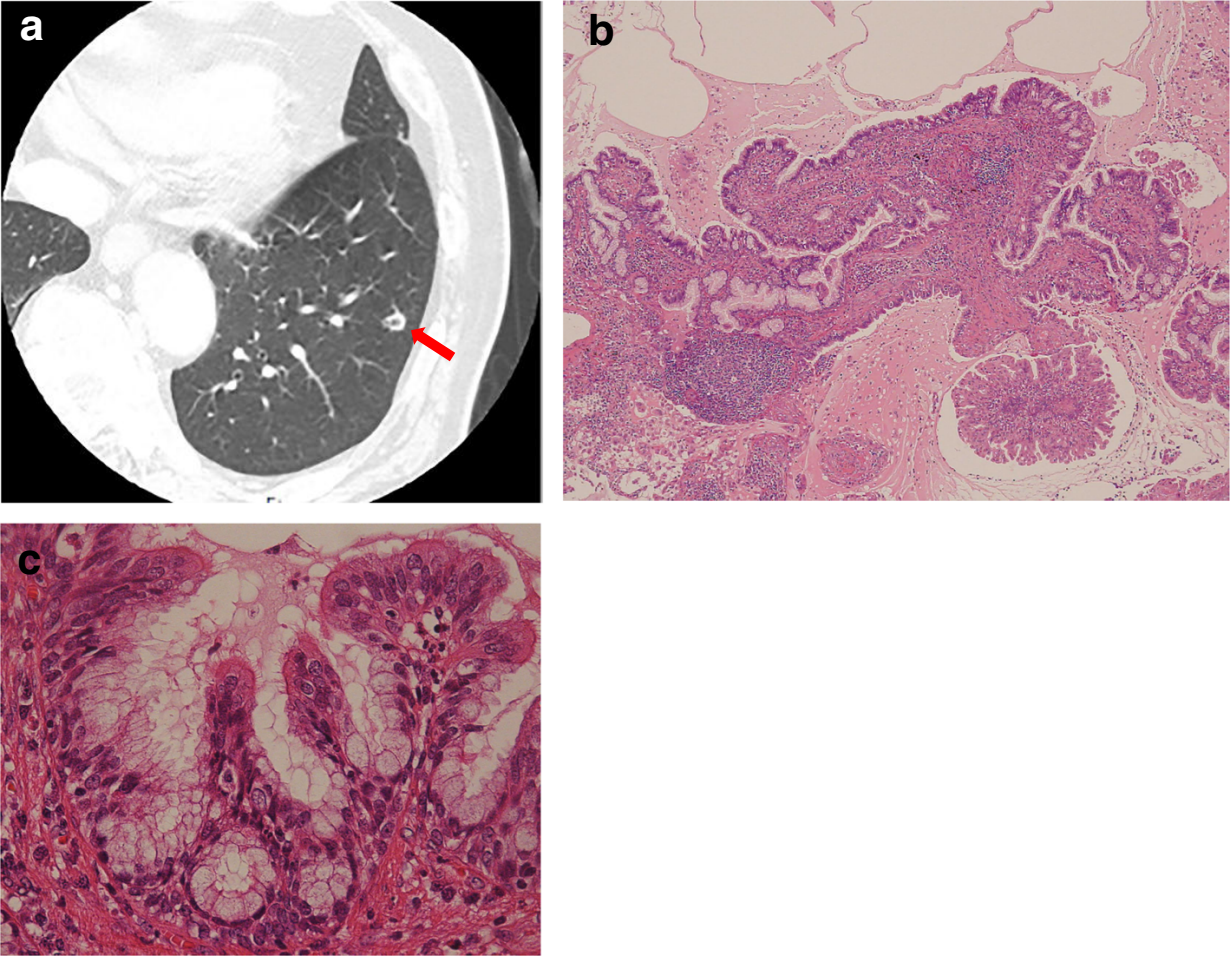
**Imaging and pathological findings in Case 1. a)** Chest *computed tomography* (CT) shows a 0.8 × 0.8-cm nodule in segment 9 of the left lung (arrow). **b)** Low-power histologic view of the resected tumor (hematoxylin-eosin staining). The tumor consists of a fibrovascular core and papillomatous fronds lined by pseudostratified columnar epithelium. **c)** High-power view of b (hematoxylin-eosin staining). The columnar epithelium consists of ciliated columnar cells, goblet cells and basal cells with no cytologic or architectural atypia.

In January 2010, a 64-year-old man with a smoking history of *two* packs of cigarettes daily for 40 years was noted to have an abnormal lesion in the left lung on chest computed tomography (CT) performed as follow-up after treatment for laryngeal cancer. He was therefore referred to our department. The previous laryngeal cancer had shown complete clinical response after chemoradiotherapy and the patient had no respiratory symptoms. Laboratory data were unremarkable, and serum tumor marker levels were all within normal limits. Chest CT revealed a solitary pulmonary nodule 0.8 × 0.8 cm in size in segment 9 of the left lung (Figure 1a). In August 2010, the patient underwent partial resection of the left lower lobe of the lung by video-assisted thoracoscopic surgery (VATS) for treatment and diagnosis. Intraoperative pathological examination using frozen sections suggested inflammatory granuloma without malignant features. Postoperative histological examination demonstrated that the tumor *was* comprised *of* a fibrovascular core and papillomatous fronds lined by pseudostratified columnar epithelium (Figure 1b). The columnar epithelium consisted of ciliated columnar cells, goblet cells and basal cells with no cytologic or architectural atypia (Figure 1c). On the basis of these morphological findings, glandular papilloma of the lung was diagnosed. As of 44 months postoperatively, the patient remains clinically and radiographically disease-free.

### Case 2

**Figure 2:**
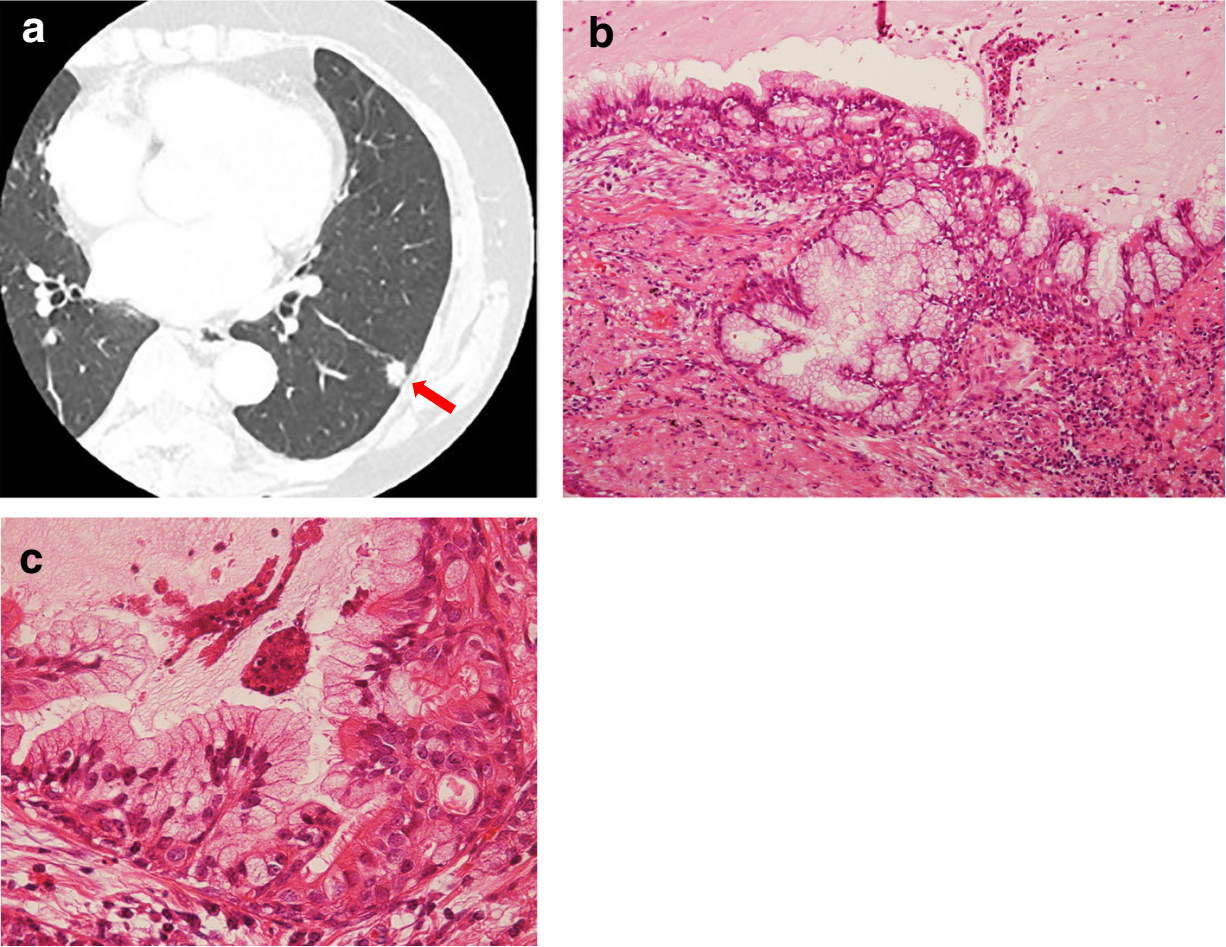
**Imaging and pathological findings in Case 2. a)** Chest *computed tomography* (CT) shows a 1.0 × 0.8-cm nodule in segment 9 of the left lung (arrowhead). **b)** Low-power histologic view of the resected tumor (hematoxylin-eosin staining). The tumor is composed of a fibrovascular core and papillomatous fronds lined by pseudostratified columnar epithelium. **c)** High-power view of b (hematoxylin-eosin staining). The pseudostratified columnar epithelium consists of ciliated columnar cells and numerous mucous cells, with no cytologic or architectural atypia.

In August 2010, a 73-year-old woman with no smoking history was noted to have an abnormal lesion in the left lung during a health check-up and was thus referred to our department. She had no relevant past history and no respiratory symptoms. Laboratory data were unremarkable, and serum levels of tumor markers were all within normal limits. Chest CT revealed a solitary pulmonary nodule with air and solid components 1.0 × 0.8 cm in size in segment 9 of the left lung (Figure 2a). In November 2010, the patient underwent VATS partial resection of the lower lobe of the left lung for treatment and diagnosis. Intraoperative pathological examination using frozen sections suggested glandular papilloma of the lung. Postoperative histological examination showed the tumor *was* comprised *of* a fibrovascular core and papillomatous fronds lined by pseudostratified columnar epithelium (Figure 2b). The pseudostratified columnar epithelium consisted of ciliated columnar cells and numerous mucous cells with no cytologic or architectural atypia (Figure 2c). As of 41 months postoperatively, the patient remains clinically and radiographically disease-free.

## Conclusions

Pulmonary papillomas can be classified according to the number of lesions, location or histology [2]. With regard to the number of lesions, pulmonary papillomas are divided into *two* types, multiple and solitary. Multiple papillomas, representing papillomatosis, are usually related to infection with papillomavirus, most often occurring in children and young adults, and involving both the upper and lower respiratory tracts. Solitary papillomas are rarer and predominantly affect adults [8-11]. According to location, pulmonary papillomas can be classified as central endobronchial or peripheral bronchiolar types. Flieder *et al*. reviewed 14 cases of solitary pulmonary papillomas, describing 13 as central endobronchial papillomas and only 1 as *a* peripheral bronchiolar papilloma [2]. The majority of peripheral bronchiolar papillomas are small, asymptomatic and only discovered incidentally on chest radiography, as in both our cases. In contrast, *a* central endobronchial papilloma often causes *a* persistent, paroxysmal and/or productive cough. Screening for lung cancer using chest radiography has recently gained popularity among healthy people in Japan, so opportunities to discover asymptomatic peripheral papillomas may increase. Pulmonary papillomas are histologically divided into *three* categories: squamous cell*,* glandular and mixed types [2,12]. Squamous cell papillomas are the most common. Glandular papillomas of the peripheral lung seem to be rare, with only 20 cases reported to date in the English literature [2,4-7]. Peripheral bronchiolar papillomas sometimes grow along alveolar walls and display an appearance similar to peripheral adenocarcinomas of the bronchioloalveolar or papillary type. For differential diagnosis, it is noteworthy that endobronchiolar papillomatous fronds are constantly present and spread along the alveolar walls is limited in alveoli adjacent to peripheral papillomas. The presence of ciliated cells and basal cells is considered an important finding for confirming the diagnosis [4].

In both of our cases, the patient was successfully treated by local excision. To the best of our knowledge, malignant transformation has only been reported with the squamous variant [13]. The glandular variant does not appear to recur locally after local excision and has no proven malignant potential. We therefore recommend local excision for solitary glandular papilloma.

## Consent

Written informed consent was obtained from the patient for the publication of this case presentation and accompanying images. A copy of the written consent is available for the review by the Editor-in-Chief of this journal.

## Abbreviations

CT: Computed tomography; VATS: Video-assisted thoracoscopic surgery.

## Competing interests

The authors declare that they have no competing interests.

## Authors’ contributions

KK and HH wrote the manuscript. KK, HH and MH performed *the* surgery. TH carried out the pathological examination. All authors approved the final manuscript.
